# Deep sequencing prompts the modification of a real-time RT-PCR for the serotype-specific detection of polioviruses

**DOI:** 10.1016/j.jviromet.2018.11.007

**Published:** 2019-02

**Authors:** Marisa Holubar, Malaya K. Sahoo, ChunHong Huang, Alisha Mohamed-Hadley, Yuanyuan Liu, Jesse J. Waggoner, Stephanie B. Troy, Lourdes García-García, Leticia Ferreyra-Reyes, Yvonne Maldonado, Benjamin A. Pinsky

**Affiliations:** a Department of Medicine, Division of Infectious Diseases and Geographic Medicine, Stanford University School of Medicine, Stanford, CA, United States; b Department of Pathology, Stanford University School of Medicine, Stanford, CA, United States; c Department of Pediatrics, Division of Infectious Diseases, Stanford University School of Medicine, Stanford, CA, United States; d Eastern Virginia Medical School, Norfolk, VA, United States; e Instituto Nacional de Salud Pública, Cuernavaca, Morelos, Mexico

**Keywords:** Poliovirus, Enterovirus c human, Poliovirus vaccine oral, High-throughput nucleotide sequencing, Real-time polymerase chain reaction

## Abstract

Polioviruses are members of the Enterovirus C species and asymptomatic fecal shedding allows for their transmission and persistence in a community, as well as the emergence of vaccine-derived polioviruses. Using three serotype-specific real-time RT-PCR (rRT-PCR) assays, the shedding and circulation of oral poliovirus vaccine (OPV) strains was previously investigated in a prospective cohort of Mexican children, their contacts, and nearby sewage. Subsequently, a deep sequencing approach targeting the P1 genomic region was applied to characterize OPV strains previously detected by rRT-PCR. Amplifiable RNA was obtained for sequencing from 40.3% (58/144) of stool samples and 71.4% (15/21) of sewage using nucleic acids extracted directly from primary rRT-PCR-positive specimens. Sequencing detected one or more OPV serotypes in 62.1% (36/58) of stool and 53.3% (8/15) of sewage samples. All stool and sewage samples in which poliovirus was not detected by deep sequencing contained at least one non-polio enterovirus C (NPEV-C) strain. To improve screening specificity, a modified, two-step, OPV serotype-specific multiplex rRT-PCR was evaluated. In stool specimens, the overall agreement between the original assays and the multiplex was 70.3%. By serotype, the overall agreement was 95.7% for OPV serotype-1 (S1), 65.6% for S2, and 96.1% for S3. Furthermore, most original rRT-PCR positive/multiplex rRT-PCR negative results were collected in the summer and fall months, consistent with NPEV-C circulation patterns. In conclusion, this deep sequencing approach allowed for the characterization of OPV sequences directly from clinical samples and facilitated the implementation of a more specific multiplex rRT-PCR for OPV detection and serotyping.

## Highlights

• Deep sequencing distinguished poliovirus from non-polio enterovirus C (NPEV-C).
• Low rRT-PCR specificity resulted in false-positive Sabin 2 in stool with NPEV-C.
• Modification of a multiplex rRT-PCR restored poliovirus serotype specificity.

## 1 Introduction

Since the World Health Assembly resolution in 1988, dramatic gains have been made towards poliomyelitis eradication. In 2017, only 22 cases of wild-type poliovirus serotype 1 were reported, all from two of the three remaining polio-endemic countries, Afghanistan and Pakistan. As of July 2018, an additional 12 cases were reported from these countries. No wild-type poliovirus cases have been reported from the third endemic country, Nigeria, since 2016. In 2015, the Global Commission for the Certification of Poliomyelitis Eradication declared wild-type poliovirus serotype 2 eradicated (Morales et al., 2016a; Global Polio Eradication Initiative, 2018). No cases of wild-type poliovirus serotype 3 have been reported since 2012 (Kew et al., 2012).

However, vaccine-derived polioviruses (VDPV) continue to threaten and complicate the polio endgame strategy. VDPV are mutated forms of the oral poliovirus vaccine (OPV) that can reacquire neurovirulence characteristic of wild-type polioviruses. Since 2005, there have been up to 8 circulating VDPV (cVDPV) outbreaks per year (Morales et al., 2016b). In 2015, outbreaks of cVDPVs were reported from 7 African and Asian countries, most of which experienced disruption of routine poliovirus vaccination programs (Morales et al., 2016a). In 2017, large cVDPV outbreaks were identified in the Syrian Arab Republic (74 cases) and the Democratic Republic of the Congo (24 cases) (Mbaeyi et al., 2018; Alleman et al., 2018)

Poliomyelitis surveillance relies heavily on acute flaccid paralysis case detection, which then prompts a diagnostic and epidemiologic investigation. However, asymptomatic shedding of polioviruses is far more common than poliomyelitis (Sutter et al., 2018). Historical studies showed that up to ninety percent of infants shed OPV after vaccination and can spread OPV to their household contacts (Benyesh-Melnick et al., 1967). Recent events illustrate the ability of polioviruses to persist in the environment. In May 2013, Israel, a country whose immunization schedule replaced OPV with inactivated poliovirus vaccine in 2005, reported a “silent outbreak” of wild-type poliovirus type 1 detected from environmental sampling. The last positive sewage sample was detected in April 2014, nearly 10 months after a massive public health response began that included supplementary vaccination campaigns (Tasher et al., 2016). Similarly, in August 2016, four cases of wild poliovirus type 1 were identified in northern Nigeria after two years with no reported cases. Further, the Nigerian polioviruses were genetically identical to strains which circulated there at least five years earlier, suggesting that silent poliovirus circulation could be sustained for prolonged periods. VDPVs exhibit similar properties. For example, Nigeria reported a cVDPV-2 case in May 2015, and in March 2016 environmental sampling also detected cVDPV-2, illustrating these viruses’ ability to persist in communities (Morales et al., 2016a). Methodology to accurately assess asymptomatic shedding and circulation in communities is critical in the post-eradication era.

Traditionally, poliovirus detection from stool or sewage requires cell culture in order to produce adequate concentrations of virus for characterization and sequencing. In 2006, the Maldonado laboratory developed real-time PCR assays that could detect OPV directly from stool. Primers for these assays were designed to distinguish non-revertant from revertant OPV strains, those which contained the characteristic point mutations in the 5′ untranslated region linked to vaccine-associated paralytic poliomyelitis (Gnanashanmugam et al., 2007; Troy et al., 2011). Using these assays, the shedding and circulation of OPV in a prospective cohort of Mexican children and their contacts was investigated. In that study, ∼6% of over 2500 stool samples were positive for OPV (Troy et al., 2014). In a subsequent study, a targeted, deep sequencing methodology was developed to detect and characterize the OPV strains shed by this cohort without virus propagation in culture (Sahoo et al., 2017). Herein, this deep sequencing approach is utilized to describe the identification of related, non-polio enterovirus C (NPEV-C) strains in stool specimens thought to contain OPV-2. This resulted in the subsequent re-evaluation of the specificity of the original OPV assays and the evaluation of a modified multiplex, real-time RT-PCR (rRT-PCR) assay for the detection and differentiation of OPV serotypes directly from stool.

## 2 Materials and methods

### 2.1 Ethics

The study protocol was approved by the following: Ethics, Biosafety, and Research Committees of the Mexican Instituto Nacional de Salud Pública and authorized and registered by the Public Health Center of Orizaba, Veracruz, Mexico; Stanford University Institutional Review Board; and Eastern Virginia Medical School Institutional Review Board.

### 2.2 Study samples

Samples were previously collected and de-identified (Troy et al., 2014). Seventy-two children and 144 household contacts were followed from four municipalities in or near Orizaba, Veracruz, Mexico in a prospective cohort study conducted from August 2010 – August 2011. Per Mexican national guidelines enacted in 2007, children receive IPV at 2, 4, 6, and 18 months of age. In addition, children 5 years old or younger who have received at least 2 doses of IPV are eligible to receive trivalent OPV during biannual National Health Weeks (NHW). At each of 12 monthly visits, beginning in August 2010, stool was collected from the enrolled child and the household contacts, and stored at -70 °C. Stool samples were tested by three serotype-specific two-step, rRT-PCR reactions that differentiate Sabin 1 (S1), 2 (S2), and 3 (S3) (Troy et al., 2011).

### 2.3 RNA extraction and cDNA generation for Sequencing

RNA was extracted from frozen stool samples on the QIAcube instrument using the RNeasy mini kit (both from Qiagen) according to the manufacturer’s instructions. Sewage samples were collected and processed as described (Troy et al., 2012). Five μL isolated RNA was reverse transcribed for 1 or 5 h by SuperScript III reverse transcriptase (Thermo Fisher Scientific Inc.) according to the manufacturer’s instructions using primer Q8R modified with a 5′ sequence tag (5′- *TACGGTAGCAGAGACTTGGTCT*AAGAGGTCTCTRTTCCACAT-3′). The ∼3.5 kb region encoding the viral structural proteins VP1 - VP4 along with the 5′ untranslated region (UTR) was amplified using the modified primer Q8R and primer UFP also modified with a 5′ sequence tag (5′-*GCGGCCGCTAATACGACTCACTATAGG*TTAAAACAGCTCTGGGGTTG) (Chumakov, 1996; Neverov and Chumakov, 2010; Boot et al., 2004). Each 50 μL reaction contained LongAmp Hot Start Taq 2X Master mix (New England BioLabs), 400 nM of each primer, 400 ng/μL BSA, and 2–4 μL of cDNA. The reactions were performed in a Applied Biosystems Veriti 96-well thermal cycler, using the following cycling parameters: 94 °C for 2 min; 40 cycles of 94 °C for 30 s, 70 °C for 1 s (ramp rate 20%), 55 °C for 45 s (ramp rate 20%), and 65 °C for 3 min 20 s; final extension for 10 min at 65 °C. The PCR products were electrophoresed on a 1% agarose gel and visualized by ethidium bromide staining. The ∼3.5 kb amplicon was excised and purified using the QIAquick Gel Extraction Kit (Qiagen). The purified PCR products were quantitated using the Qubit dsDNA HS Assay Kit (Thermo Fisher Scientific Inc.) according to the manufacturer's instructions, and stored in −20 °C prior to library preparation and sequencing (Supplemental Materials and as previously described (Sahoo et al., 2017)).

### 2.4 Alignment

FASTQ files were aligned to full-length S1, S2, and S3 reference sequences (GenBank: V01150.1, AY184220.1, and AY184221.1, respectively) using Burrows-Wheeler Aligner's Smith-Waterman Alignment (BWA-SW) with default parameters (Li and Durbin, 2010). The BAM alignment files were checked visually using Tablet for coverage and count of reads aligned to each reference (Milne et al. (2013)). An OPV serotype was considered detected if at least 100X coverage was obtained over 95% of the 5′UTR and VP1-4 region. FASTQ files were also aligned to representative enterovirus C reference sequences and detection of NPEV-C viruses was achieved through evaluation of the sequences encoding the structural proteins VP1-4 (Table 1). An NPEV-C strain was considered detected if at least 100X coverage was obtained over 95% of the VP1-4 region. Potential recombinants were identified by evaluating the distribution of aligned reads and their start and end points as described (Chen et al., 2008).

**Table 1:** Enterovirus C Reference Sequences and genomic regions used for detection by deep sequencing.

Name	Genbank Accession	Start^a^	End^b^
Sabin 1 (S1)	V01150	743	3386
Sabin 2 (S2)	AY184220	748	3385
Sabin 3 (S3)	AY184221	743	3377
Coxsackie A1 Tompkins	AF499635	712	3337
Coxsackie A19	AB828290	338	2972
Coxsackie A22 Chulman	AF499643	716	3347
Enterovirus C104 AK11	AB686524	718	3349
Enterovirus C105 PER153	JX393302	636	3264
Enterovirus C109 L87	JN900470	222	2853
Enterovirus C113 BBD83	KC344834	687	3318
Enterovirus C116	JX514942	690	3324
Enterovirus C117 LIT22	JX262382	673	3304
Enterovirus C118 PER161	JX393301	623	3254

a Defined as position 1 of VP4.

b Defined as position 1 of P2A.

### 2.5 Multiplex two-step rRT-PCR assay

Serotype-specific poliovirus primers and probes targeting the VP1 gene were adopted from (Kilpatrick et al. (2009)) except for the Sabin 3 probe which was adopted from (Taniuchi et al. (2015)). Reverse transcription utilized the SuperScript III Reverse Transcriptase (Thermo Fisher Scientific Inc.) and otherwise was performed as previously described (Troy et al., 2011). Real-time PCR was performed in a total volume of 20 μL, and 5 μL of cDNA and 10 μL of 2X IQ Multiplex Powermix (BioRad) were added to each reaction. The primer and probe concentrations in the final reaction mixture are listed in Table 2. Amplification and detection was performed on the CFX384 Real-Time System (BioRad). Cycling conditions were the following: 52 °C for 2 min; 95 °C for 10 min; 40 cycles of 95 °C for 15 s and 60 °C for 60 s. Detection was performed in channels 1 (515–530 nM), 3 (610–650 nM), and 4 (675–690) at 60 °C using 6-carboxyfluorescein, Texas Red, and Cy5 labeled probes, respectively. The threshold was set at 300 RFU in all channels. Samples were run in triplicate, and a sample was considered positive if 2 out of 3 reactions crossed the cycle threshold (C_T_) in less than 37 cycles.

**Table 2:** Primer and Probe Sequences for the Multiplex RT-PCR.

Name	Sequence (5' → 3')	Concentration
S1 Forward	AGGTCAGATGCTTGAAAGC	300nM
S1 Reverse	CCACTGGCTTCAGTGTTT	300nM
S1 Probe	Cy5-TTGCCGCCCCCACCGTTTCACGGA-BHQ2	125nM
S2 Forward	CCGTTGAAGGGATTACTAAA	300nM
S2 Reverse	CGGCTTTGTGTCAGGCA	300nM
S2 Probe	6FAM-ATTGGTTCC-ZEN-CCCGACTTCCACCAAT-IBFQ	150nM
S3 Forward	AGGGCGCCCTAACTTT	400 nM
S3 Reverse	TTAGTATCAGGTAAGCTATC	400 nM
S3 Probe	TexasRed-X-TCACTCCCGAAGCAACAG-BHQ2	300nM

6FAM, 6-carboxyfluorescein; Texas Red-X, Texas Red-X NHS Ester, BHQ2, Black Hole Quencher-2; ZEN, internal quencher; IBFQ, Iowa Black Fluorescent Quencher.

Reference and control materials, and methods for evaluating cross-reactivity, linearity, and lower limit of detection can be found in the Supplemental Materials.

## 3 Results

### 3.1 Detection of oral poliovirus vaccine strains in stool by deep sequencing

To characterize the OPV strains fecally shed by vaccinated children and close contacts, two-step RT-PCR targeting the poliovirus 5′ UTR and P1 region, encoding the structural viral proteins VP1-4, was carried out. Extracted RNA that had previously tested positive by serotype specific rRT-PCR assays was used as template The virus was not cultured prior to two-step RT-PCR, resulting in 40.3% (58/144) of stool samples with amplifiable RNA. These amplicons were utilized to generate fragmentation libraries for evaluation by deep sequencing.

A median of 629,100 reads per sample (range 62,090–5,811,320; inter quartile range 825,907) was obtained, and one or more OPV serotypes were detected in 62.1% (36/58) of samples. Furthermore, there was 83.3% (30/36) detection of the predominant OPV serotype as determined by rRT-PCR (Troy et al., 2011). In samples where more than one serotype was detected by rRT-PCR, deep sequencing detected minor serotypes in 50.0% (9/18). Overall, deep sequencing detected 75.4% (46/61) of the polioviruses identified by rRT-PCR, including 73.3% (11/15) of S1, 73.9% (17/23) of S2, and 78.2% (18/23) of S3. Sequencing detected but called different serotypes in two samples and identified additional serotypes not detected by rRT-PCR in three samples (Table S3). Interestingly, no poliovirus was detected in 37.9% (22/58) of samples by this sequencing approach, despite the presence of appropriately sized amplification products and the detection of S2 by rRT-PCR.

### 3.2 Detection of oral poliovirus vaccine strains in sewage

To characterize the OPV strains that persisted in the environment, sewage samples previously positive by serotype specific rRT-PCR assays were evaluated by deep sequencing. As with the stool samples, the virus was not cultured prior to two-step RT-PCR, resulting in 71.4% (15/21) of sewage samples with sequencable RNA. A median of 540,938 reads per sample (range 130,577 – 2,604,416; inter quartile range 537,606) was obtained. One or more OPV serotypes were detected in 53.3% (8/15) of sewage samples, and overall, deep sequencing detected 41.2% (7/17) of the polioviruses identified by rRT-PCR, including 100% (1/1) of S1, 40.0% (6/15) of S2, and 0.0% (0/1) of S3. In one sample, S1 was detected by sequencing but S2 was detected by rRT-PCR. Similar to the stool samples, no poliovirus was detected in 46.7% (7/15) of samples by this sequencing approach, despite appropriately sized P1 amplification products and adequate reads per sample.

### 3.3 Identification of non-polio enterovirus C strains in stool and sewage

It was suspected that the stool and sewage samples without poliovirus by sequencing contained closely related NPEV-C strains. To test this hypothesis, reads from all samples were aligned to reference enterovirus C sequences (Table 1). All stool samples in which sequencing did not detect poliovirus contained one or more NPEV-C strains, including ten with coxsackievirus A1 (CV A1), eight with coxsackievirus A19 (CV A19), three with both CV A1 and CV A19, and one with enterovirus C116 (EV C116). Furthermore, 5 stool samples in which a poliovirus was identified by sequencing also contained additional NPEV-C RNA, including samples with CV A1 (one), CV A19 (two), or both CV A1 and CV A19 (two). Similarly, all sewage samples in which sequencing did not detect poliovirus contained one or more NPEV-C strains, including one with CV A1, one with EV C116, one with CV A1 and EV C116, two with CV A19 and EV C116, and two samples containing all three strains, CV A1, CV A19, and EV C116. The sewage samples in which OPV was detected by sequencing also contained NPEV-C strains (7/8), including three with CV A1, two with CV A1 and EV C116, one with CV A1 and CV A19, and one with CV A1, CV A19, and EV C116. No alignment was observed to enterovirus A, B, or D species. Furthermore, no NPEV-C/Sabin recombinants were identified.

### 3.4 Cross-reactivity of the Original Sabin 2 rRT-PCR assay

The original S2 rRT-PCR assay was positive in all stool and sewage samples from which only NPEV-Cs were detected by deep sequencing, suggesting a lack of specificity. Though the S2 rRT-PCR assay did not detect oligonucleotides encoding CV A1 5′UTR sequences at concentrations up to 5 × 10^8^ copies/reaction, this assay detected CV A19 and EV C116 oligonucleotides at concentrations as low as 5 copy/reaction. No amplification was observed in the S1 or S3 assays.

### 3.5 Multiplex rRT-PCR assay analytical evaluation

The lack of specificity of the original S2 assay prompted a new approach to detect OPV serotypes. A two-step, serotype-specific multiplex rRT-PCR was adapted from the literature (Kilpatrick et al., 2009; Taniuchi et al., 2015) and an analytical evaluation performed. Using serial dilutions of oligonucleotides containing the target region for each Sabin serotype, the linear range for each serotype extended from 10^1^ to 10^8^ cDNA equivalents/μL. Based on standard curves generated using these oligonucleotides the concentration of the cultured Sabin reference strains was estimated and expressed in poliovirus copies/reaction. Using serial dilutions of Sabin RNA the 95% LLOD for each serotype was calculated to be: 3 copies/reaction for S1, 4 copies/reaction for S2, and 3 copies/reaction for S3.

In contrast to the original S2 assay, the multiplex rRT-PCR assay did not detect oligonucleotides encoding CV A19 and EV C116 VP1 sequences in any channel, even at concentrations as high as 5 × 10^8^ copies/reaction.

### 3.6 Agreement between original and multiplex rRT-PCR assays

To compare assay performance on clinical samples, 256 previously tested stool samples were re-tested using the multiplex rRT-PCR assay, including all 144 samples that were positive for at least one serotype using the original rRT-PCR assays (Table 3). Negative samples were selected at random. Archived RNA stored at −80 °C was used for 229 of the samples (117 positive, 112 negative using the original rRT-PCR assay); 27 samples (all positive using the original rRT-PCR assay) required new RNA extraction. For the presence of any OPV strain, the positive agreement between the original and multiplex rRT-PCR assays was 46.2%, and the overall agreement 70.3% (Table 4). This lack of agreement was due primarily to S2, which demonstrated a positive agreement of just 27.6% and an overall agreement of 65.6%.

**Table 3:** Comparison of the multiplex rRT-PCR assay with the single-serotype rRT-PCR assays for detection of (A) any serotype, (B) Sabin 1 (S1), (C) Sabin 2 (S2), and (D) Sabin 3 (S3).

A				
		Any Serotype		
		S1, S2, or S3 rRT-PCR		
		Positive	Negative	Total
Multiplex rRT-PCR	Positive	68	0	68
	Negative	76	112	188
	Total	144	112	256

B				
		Sabin 1		
		S1 rRT-PCR		
		Positive	Negative	Total
Multiplex rRT-PCR	Positive	27	7	34
	Negative	4	218	222
	Total	31	225	256

C				
		Sabin 2		
		S2 rRT-PCR		
		Positive	Negative	Total
Multiplex rRT-PCR	Positive	29	12	41
	Negative	76	139	215
	Total	105	151	256

D				
		Sabin 3		
		S3 rRT-PCR		
		Positive	Negative	Total
Multiplex rRT-PCR	Positive	47	6	53
	Negative	4	199	203
	Total	51	205	256

**Table 4:** Positive and negative percent agreement between the multiplex and single-serotype rRT-PCR assays.

Serotype	Positive % agreement	Negative % agreement	Overall % agreement	Kappa
Any Serotype	46.2	100.0	70.3	0.439 (0352-0.527)
Sabin 1 (S1)	87.1	96.9	95.7	0.806 (0.695-0.917)
Sabin 2 (S2)	27.6	92.1	65.6	0.217 (0.112-0.321)
Sabin 3 (S3)	92.2	97.1	96.1	0.879 (0.806-0.952)

### 3.7 Shedding of oral poliovirus vaccine strains in relation to national health weeks

**Fig. 1:**
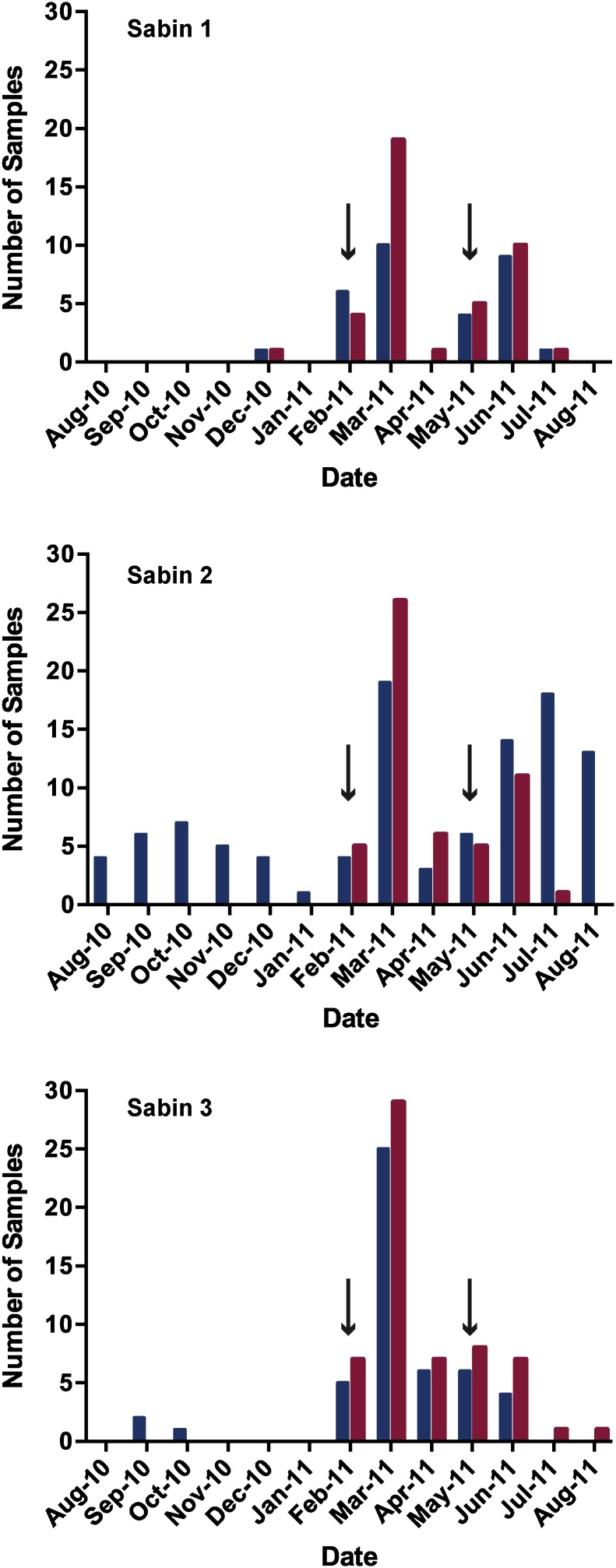
rRT-PCR Positive stool specimens by month of collection. Results from the original rRT-PCR assays are represented by the blue bars, whereas the results from the multiplex rRT-PCR assay are represented by the red bars. Sabin 1 (S1), 2 (S2), and 3 (S3) are displayed separately. Black arrows indicate national health weeks (For interpretation of the references to colour in this figure legend, the reader is referred to the web version of this article).

During the study period there were two national health weeks (NHWs), 2/15/11−2/19/11 and 5/28/11 − 6/3/11. The number of positive stool samples for each serotype that were detected with the original and multiplex rRT-PCRs was plotted versus the month in which the specimens were collected (Fig. 1). For S1 and S3, both the original and multiplex assays showed similar patterns (Fig. 1A and C), detecting a majority of positive stool specimens between February and July 2011, consistent with the timing of the NHWs. For S2, however, positive stool specimens were identified by the original rRT-PCR throughout the year, including the fall of 2010, when no OPV strains were detected using the multiplex assay (Fig. 1B). Furthermore, the multiplex assay detected the shedding of S2 in stool with timing similar to S1 and S3, and consistent with the NHWs.

## 4 Discussion

This study describes the application of a targeted deep sequencing method to characterize OPV shed in the stool of vaccinated children, their close contacts, and sewage, collected in vaccinated communities in Mexico that had previously been identified as OPV positive by rRT-PCR. Of the sequenced samples, no OPV strains were identified in 38.1% of stool and 50% of sewage specimens. S2 detection by the rRT-PCR assay drove these discrepant results and NPEV-C strains were sequenced from these samples. These findings prompted the adaptation of a two-step, serotype-specific multiplex rRT-PCR with preserved sensitivity and improved specificity for detecting OPV directly from stool and sewage samples.

Several plausible explanations for the discrepancy between the S2 rRT-PCR assay and the deep sequencing methodology were investigated. Based on the findings presented here, the most likely explanation is that the S2 rRT-PCR assay lacks specificity for OPV, and also detects the presence of NPEV-C strains. The Maldonado laboratory developed rRT-PCR assays to detect OPV directly from stool and distinguish non-revertant from revertant OPV strains in 2006 (Gnanashanmugam et al., 2007; Troy et al., 2011). Primers and probes targeting the 5′ untranslated region of interest were developed using sequences available in GenBank. At that time there were a total of 245 human enterovirus C 5′UTR sequences in the database, and no *in silico* cross-reaction was identified with the primers and probes used in these assays. While the number of human enterovirus C 5′UTR sequences housed in GenBank has increased ∼3.4-fold since the original rRT-PCR assay was designed (834 as of December 09, 2016) the original S2 primers and probe have mismatches to all deposited CV A1, CV A19, or EV C116 sequences and would not be predicted to amplify these strains. Though laboratories must remain vigilant for changes in assay performance and attempt to stay up to date on the availability of new sequences that may impact sensitivity or specificity (Van Leer-Buter et al., 2016), these results highlight the value of wet lab testing in determining assay performance characteristics.

It remains possible that the S2 rRT-PCR assay may have detected very low concentrations of S2 in mixed samples that were not detected via sequencing. Though the S2 rRT-PCR assay was the most sensitive of the 3 original serotype specific assays (Troy et al., 2011), this assay is ∼30-fold less analytically sensitive than the S2 component of the multiplex. Furthermore, the multiplex detected more positive samples than the original rRT-PCR assays in March 2011 shortly after the National Health Week when vaccinees and their contacts are most likely to shed OPV of all 3 serotypes in their stool. Taken together, these data indicate that the multiplex is more sensitive than the original rRT-PCR assay, not less.

The performance of the multiplex also suggests that the lack of specificity in the original S2 rRT-PCR assay drove the discrepancies between the rRT-PCR and sequencing results. The multiplex did not detect oligonucleotides representing CV A19 and EV C116 sequences and confirmed only 27% of the original S2 rRT-PCR positive results from stool samples. In addition, stool samples obtained soon after a NHW were more likely to have detectable RNA by the multiplex than later samples. Endemic NPEV-C viruses exhibit seasonal patterns of incidence, with infection being more common in summer and early autumn in temperate climates like this study site (Pons-Salort et al., 2015). The original S2 rRT-PCR results reflect this pattern with most rRT-PCR positive/multiplex negative results obtained in the summer and fall months (Fig. 1B).

The lack of specificity of the S2 rRT-PCR has varied implications for the interpretation of the findings of previous studies using the original rRT-PCR assays (Troy et al., 2011, 2014; Troy et al., 2012). Given that the overall rate of shedding was low in all studies, 2.4% in the pilot (Troy et al., 2011) and 6% in the larger prospective study (Troy et al., 2014), the re-classification of the S2 false positives would further reduce the rate of shedding and would likely have limited impact on the overarching conclusions of these studies. For example, evaluation of both the original assay and the multiplex rRT-PCR revealed that shedding of OPV2 by household contacts was most likely the source of infection of non-vaccinated children and subjects older than 5 years of age living in the same household (Ferreyra-Reyes et al., 2017).

However, the conclusion that S2 circulates longer and is transmitted more readily than S1 or S3 after NHWs is likely artefactual due to the mis-identification of NPEV-C strains as S2 (Troy et al., 2014).

This study is limited by the number of samples that did not contain amplifiable viral nucleic acids for sequencing, due in part to the large size of the amplicon target (∼3.5 kb) and the low levels of virus in primary specimens. Nevertheless, this sequencing approach allowed the unexpected identification of NPEV-C strains, and as such, facilitated the implementation of the more specific multiplex rRT-PCR for future studies of OPV shedding. In addition, this sequencing approach was not designed to detect the OPV-NPEV-C recombinants that contribute to cVDPV outbreaks, which are typically composed of vaccine-derived P1 sequences with genes encoding the nonstructural proteins derived from an NPEV-C strain (Burns et al., 2014; Kew et al., 2005; Jegouic et al., 2009; Riquet et al., 2008). Though samples that contained both OPV and NPEV-C strains were identified, Sabin serotype and NPEV-C strain specific amplification primers may be necessary to improve sequence detection of mixed infections. Future whole genome approaches from primary specimens that evaluate the 5′UTR and P1, as well as the non-structural genes, will be required to detect recombinants and study their contribution to the development of VDPVs.

In conclusion, this work describes the application of a targeted, deep sequencing methodology to detect and characterize the OPV strains directly from clinical samples without culture enrichment. The application of this sequencing method resulted in the unexpected detection of NPEV-C strains in stool specimens thought to be positive for S2, and prompted the modification and evaluation of a multiplex, real-time RT-PCR assay for the serotype-specific detection of polioviruses. Surveillance for asymptomatic shedding and circulation remains a critical component of poliovirus eradication efforts.

## Financial support

Financial support for this study was provided by the Bill and Melinda Gates Foundation (YM) and the Stanford Child Health Research Institute (BAP).

## Potential conflicts of interest

None.
