# Supplementary material for: Deep sequencing prompts the modification of a real-time RT-PCR for the serotype-specific detection of polioviruses
Source: J Virol Methods. 2019 Feb;264:38–43. doi: 10.1016/j.jviromet.2018.11.007 (PMC6320388; doi:10.1016/j.jviromet.2018.11.007)
Supplement: Supplementary file 1 [file mmc1.docx]

**Supplemental Data File**

**Deep Sequencing Prompts the Modification of a Real-time RT-PCR for the Serotype-Specific Detection of Polioviruses**

Marisa Holubar^&^, Malaya K. Sahoo^&^, ChunHong Huang, Alisha Mohamed-Hadley, Yuanyuan Liu, Jesse J. Waggoner, Stephanie B. Troy, Lourdes García-García, Leticia Ferreyra-Reyes, Yvonne Maldonado, and Benjamin A. Pinsky

^&^These authors contributed equally.

**Library preparation**

Fragment Libraries were prepared from approximately 50 ng of PCR products using NEBNext DNA Library Prep reagents for Illumina (New England BioLabs) as per manufacturer’s instructions. DNA was fragmented using NEBNext dsDNA Fragmentase reagents, supplemented with 15 mM MgCl_2_ and incubated at 37°C for 30 min. The fragmentation reaction was stopped by adding 5 μL of 0.5M EDTA (Ambion) and fragments were purified using Agencourt AMPure XP magnetic beads (Beckman Coulter). Purified fragments were repaired and dA tailed using NEBNext Ultra End Repair-dA-Tailing module. NEBNext adaptor for Illumina was ligated to the dA-tailed fragments using NEBNext Ultra Ligation module and the adaptor was linearized by Uracil-Specific Excision Reagent Enzyme following the manufacturer’s instructions. Adaptor ligated fragments were purified using AMPure XP magnetic beads and the library was enriched by PCR using NEBNext High-fidelity PCR master mix and NEBNext multiplex oligos for Illumina. The following cycling conditions were used: 98°C for 10 sec, 12 cycles of 98°C for 10 sec, 65°C for 30 sec, and 72°C for 30 sec. This step also adds sample specific barcodes.

The resulting PCR products were purified using AMPure XP magnetic beads and checked for quality on the Agilent 2100 Expert Bioanalyzer using the high sensitivity DNA kit (Agilent Technologies). The Bioanalyzer concentration estimate was used for normalization of each sample library in the final pool of 24 samples for a single sequencing run. To eliminate any remaining adaptors or primer-dimers, the pooled library was purified using AMPure XP magnetic beads. Washing steps used a lower ethanol concentration (70%). The final library pool was checked for quality on the Bioanalyzer as above, and the library concentration was estimated using the Qubit dsDNA HS Assay Kit. The library concentration was calculated using conversion factor 1 ng/μL = 6 nM per Illumina guidelines for libraries with ~250 bp-sized fragments. The libraries were adjusted to concentrations of 4 nM before using downstream.

**Illumina Sequencing**

The pooled library was denatured using 0.2N sodium hydroxide (Sigma-Aldrich), diluted to 3pM with hybridization buffer (Illumina Inc.) and mixed 80:20 with denatured 3pM PhiX. The library was loaded into the Illumina flowcell and 150 cycle paired end sequencing was performed using MiSeq Reagent kit V2 reagents on a MiSeq Sequencer with MiSeq Control software version 2.4.1.3 (all from Illumina Inc.). FASTQ files were generated using the MiSeq reporter software (version 2.4.60.80).

**Reference and Control Materials**

Sabin serotype 1, 2, and 3 vaccine strains were cultured and RNA extracted as described (1). Briefly, the vaccine strains were obtained from the laboratory of Dr. Konstantin Chumakov at the United States Food and Drug Administration (FDA), and were prepared and titrated according to the World Health Organization (WHO) Poliovirus Laboratory Manual (World Health Organization, Geneva, Switzerland, 4^th^ Edition, 2004). Single-stranded DNA (ssDNA) oligonucleotides (Integrated DNA Technologies) were designed using Geneious 6.0.6 software (Biomatters) and encode the 5’ UTR and VP1 genomic regions for coxsackievirus A1 (CV A1) and A19 (CV A19), as well as enterovirus C116 (EV C116) that align to the targets of the original rRT-PCR assays (1) and newly constructed multiplex rRT-PCR, respectively (Table S1). A dsDNA composite oligonucleotide encoding the Sabin 1, Sabin 2, and Sabin 3 VP1 nucleotide sequences targeted by the multiplex rRT-PCR was also obtained (Integrated DNA Technologies, Table S2) and utilized as calibrator to quantitate the cultured Sabin reference strains.

**Cross-Reactivity, Linearity, and Lower Limit of Detection**

To evaluate assays for NPEV-C cross-reactivity, CV-A1, CV-A19, and EV-C116 ssDNA oligonucleotides were tested using 10-fold dilution series.

Linearity studies for the multiplex rRT-PCR assay were performed on serial 10-fold dilutions of Sabin 1, Sabin 2, and Sabin 3 composite dsDNA. Dilutions from 8.0 log_10_ copies/μL to 1 copy/μL were tested in triplicate on 7 plates. The linear range was established by fitting a best-fit line to the data by ordinary least squares regression and included the range where the R^2^ value for this line was ≥ 0.95.

RNA from the cultured Sabin reference strains was used to establish the serotype-specific lower limit of 95% detection (95% LLOD) of the multiplex rRT-PCR assay. Seven replicates for Sabin 1 and 2 and six replicates for Sabin 3 of eight 2-fold dilutions were tested on three runs. The 95% LLOD was then calculated using probit analysis.

**REFERENCES**

1. Troy SB, Ferreyra-Reyes L, Huang C, et al. Use of a novel real-time PCR assay to detect oral polio vaccine shedding and reversion in stool and sewage samples after a mexican national immunization day. *J Clin Microbiol.* 2011;49:1777-1783.

**Table S1**. Synthetic single-stranded DNA Oligonucleotides encoding Non-Polio Enterovirus C sequences

| **Target** | **Sequence (5' → 3')** |
| --- | --- |
|  |  |
| 5’UTR |  |
|  |  |
| Coxsackie A1 | CCCCTGAATGCGGCTAATCCTAACCATGGAGCAAGTGCCCACAAGCCAGTGGGTGGCTTGTCGTAATGCGCAAGTCTATGGCGGAACCGACTACTTTGGGTGTCCGTGTTTCCTTTTATTCTTATTATGGCTGCTTATGGTG |
|  |  |
| Coxsackie A19 | CCCCTGAATGCGGCTAATCCTAACCACGGAGCAAGTGCCCACAAACCAGTGAGTGGCTTGTCGTAACGCGCAAGT CTGTGGCGGAACCGACTACTTTGGGTGTCCGTGTTT CCTTTTATTTTTATCATGGCTGCTTATGGTG |
|  |  |
| Enterovirus C116 | CCCCTGAATGCGGCTAATCCTAACCACGGAGCAAGTGCTCACAAACCAGTGAGTGGCTTGTCGTAACGCGTAAGTCTGTGGCGGAACCGACTACTTTGGGTGTCCGTGTTTCCT TTTATTTTCATTATGGCTGCTTATGGTG |
|  |  |
| VP1 |  |
|  |  |
| Coxsackie A1 | GGATTGGGCGATTCTATTGAGGCTGCCATTGACAGCATCACACAAAATGCACTAACCACTGTACAAAATACAACACAATCAGGACCTACTCATTCAAAAGAAGTTCCAGCATTAACAGCA |
|  |  |
| Coxsackie A19 | GGAATTGATGATGTCATTGACACAGTCGTAGCCAATGCACTTAAAGTATCCATGCCACAAGTGCAAGACACACAATCAAGTGGACCAGTCAACTCAAAAGAAGTCCCTGCATTAACTGCT |
|  |  |
| Enterovirus C116 | GGGATAGAAGACACGATTGAGAAGGTAGTTGGAGATGCACTTAGAGTTTCAATGCCACAAGTTGCAAATACATCTGCATCTGGTCCAGTGAATTCCAAAGAAGTCCCCGCACTGACTGCA |
|  |  |

**Table S2**. Synthetic double-stranded DNA Oligonucleotides encoding Sabin Sequences

| **Target** | **Sequence (5' → 3')** |
| --- | --- |
|  |  |
| VP1 |  |
|  |  |
| Composite S1, S2, S3 | CACAGGGGTTAGGTCAGATGCTTGAAAGCATGATTGACAACACAGTCCGTGAAACGGTGGGGGCGGCAACGTCTAGAGACGCTCTCCCAAACACTGAAGCCAGTGGACCAGCACACTGAGGGGGCCGTTGAAGGGATTACTAAAAATGCATTGGTTCCCCCGACTTCCACCAATAGCCTGCCTGACACAAAGCCGAGCGGTCCAGCGAAGTTGCACAGGGCGCCCTAACTTTGTCACTCCCGAAGCAACAGGATAGCTTACCTGATACTAAGGCCAGTGGC |
|  |  |

**Table S3.** Discrepancies between the sequenced stool samples and the original rRT-PCR assays

| OPV Serotype(s) | |  |
| --- | --- | --- |
| Sequencing | rRT-PCR | Number |
|  |  |  |
| Sabin 1 | Sabin 2 | 1 |
| Sabin 1 | Sabin 1, 2 | 1 |
| Sabin 1 | Sabin 1, 2, 3 | 2 |
| Sabin 2 | Sabin 1, 2 | 1 |
| Sabin 2 | Sabin 2, 3 | 1 |
| Sabin 2 | Sabin 1, 2, 3 | 1 |
| Sabin 3 | Sabin 2 | 1 |
| Sabin 3 | Sabin 1, 3 | 1 |
| Sabin 3 | Sabin 2, 3 | 1 |
| Sabin 1, 2 | Sabin 2 | 2 |
| Sabin 1, 2 | Sabin 1, 2, 3 | 1 |
| Sabin 2, 3 | Sabin 1 | 1 |
| Sabin 2, 3 | Sabin 2 | 3 |
| None | Sabin 2 | 22 |
|  |  |  |
